# Novel Minimally Invasive Surgical Approaches to Endometriosis and Adenomyosis: A Comprehensive Review

**DOI:** 10.3390/jcm13226844

**Published:** 2024-11-14

**Authors:** Flávia Ribeiro, Hélder Ferreira

**Affiliations:** 1 Department of Gynecology, Minimally Invasive Gynecological Surgery Unit, Unidade Local de Saúde de Santo António, 4050-342 Porto, Portugal; 2 Instituto de Ciências Biomédicas Abel Salazar, Universidade do Porto, 4050-313 Porto, Portugal

**Keywords:** adenomyosis, endometriosis, hysteroscopy, laparoscopy, minimally invasive surgery, novel surgical technique, robotic surgery

## Abstract

Endometriosis and adenomyosis are chronic gynecological conditions that significantly impact women’s quality of life, leading to symptoms such as pelvic pain, dysmenorrhea, and infertility. Despite ongoing research, a definitive cure for these conditions remains elusive, and treatment often focuses on managing symptoms. Minimally invasive surgery is considered the gold standard for surgical management, but novel surgical techniques are continuously being developed to enhance outcomes. These innovations aim to reduce disease recurrence, improve fertility rates, and provide better long-term symptom relief. In addition, techniques like robot-assisted laparoscopy (RAS) have revolutionized the treatment of complex cases, such as deep infiltrating endometriosis (DIE), offering improved precision and effectiveness. This review explores the latest advancements in surgical approaches, their clinical efficacy, and future directions, emphasizing the need for individualized multidisciplinary care to optimize patient outcomes.

## 1. Introduction

Endometriosis and adenomyosis are gynecological conditions characterized by the abnormal presence of endometrial-like tissue outside the uterus and within the uterine muscle, respectively. Endometriosis affects approximately 10–15% of women; however, the prevalence of adenomyosis is still uncertain, ranging from 9 to 62% [1,2,3,4]. These conditions often lead to chronic pelvic pain, dysmenorrhea, infertility, and a significant reduction in quality of life. Women with endometriosis may also experience gastrointestinal and urinary tract-related pain, referred pain, nerve entrapment, and neuropathic pain [5].

Surgical treatments, such as hysterectomy or excision of endometriotic lesions, have long been considered the standard approach for managing advanced cases. However, in recent years, advancements in minimally invasive surgery and novel techniques have offered better outcomes with fewer complications and shorter recovery times [6]. The laparoscopic approach is still considered the gold standard in the management of endometriosis [7]. However, robot-assisted surgery, such as the use of the Da Vinci Surgical System, has revolutionized the precision in treatment, especially in cases of deep infiltrating endometriosis (DIE). It features jointed instruments, tremor control, and three-dimensional (3D) stereoscopic vision, enabling precise tissue visualization and manipulation. This allows for complex dissections in difficult-to-reach areas, such as the pelvic sidewalls or rectovaginal septum, with smaller incisions and fewer complications [8,9]. To minimize damage to critical nerves, nerve-sparing techniques have emerged, helping to prevent complications such as bladder dysfunction, bowel issues, and sexual dysfunction [10].

Adenomyosis traditionally required hysterectomy as the primary surgical solution. However, with advances in technology and a growing focus on fertility preservation, several surgical techniques have emerged to relieve symptoms, preserve the uterus, and improve fertility outcomes: uterine-sparing surgery, or adenomyomectomy, allows for the removal of adenomyotic lesions while preserving the uterus. The most common approach is wedge resection, which involves excising the adenomyotic tissue in a wedge-like fashion and then suturing the remaining healthy uterine tissue. This technique is particularly effective in treating adenomyomas, where the disease is localized to specific areas. For diffuse adenomyosis, the triple-flap method, where three flaps of uterine tissue are created to excise the diseased areas and then reassemble the uterus, is a possible approach. Adenomyomectomy may also be performed through minimally invasive approaches, such as laparoscopy and robotic surgery [11,12]. Other techniques are less invasive, such as High-Intensity Focused Ultrasound (HIFU), which destroys adenomyotic tissue through focused ultrasound waves, without damaging the surrounding healthy tissue. It is performed under MRI or ultrasound guidance, ensuring accurate targeting of the affected areas [13]; radiofrequency ablation (RFA) is another minimally invasive option that is being explored for adenomyosis treatment. RFA uses heat generated by radiofrequency waves to destroy adenomyotic tissue [14]. Endometrial ablation is a less invasive technique used to treat adenomyosis, particularly when the disease is limited to the inner lining of the uterus. This procedure involves destroying the lining of the uterus using heat, cold, or radiofrequency energy and is generally not recommended for women who wish to maintain their fertility [15]. Another established technique is Uterine Artery Embolization (UAE), which blocks blood flow to areas affected by adenomyosis and shrinks the adenomyotic tissue. It is a suitable option for women seeking to avoid more invasive surgeries without prioritizing fertility preservation [16].

This article aims to delve into the latest advancements in surgical techniques for treating endometriosis and adenomyosis, focusing on their clinical efficacy and future directions. The discussion will highlight the importance of individualized multidisciplinary care to optimize patient outcomes. By exploring the most recent innovations, such as uterine-sparing surgeries, minimally invasive procedures, and various ablation methods, we will provide a comprehensive overview of how these advancements are enhancing symptom relief, preserving the uterus, and improving fertility outcomes. This review underscores the shift towards more personalized treatment approaches, helping to meet the unique needs of each patient while maximizing their quality of life.

## 2. Materials and Methods

**Figure 1:**
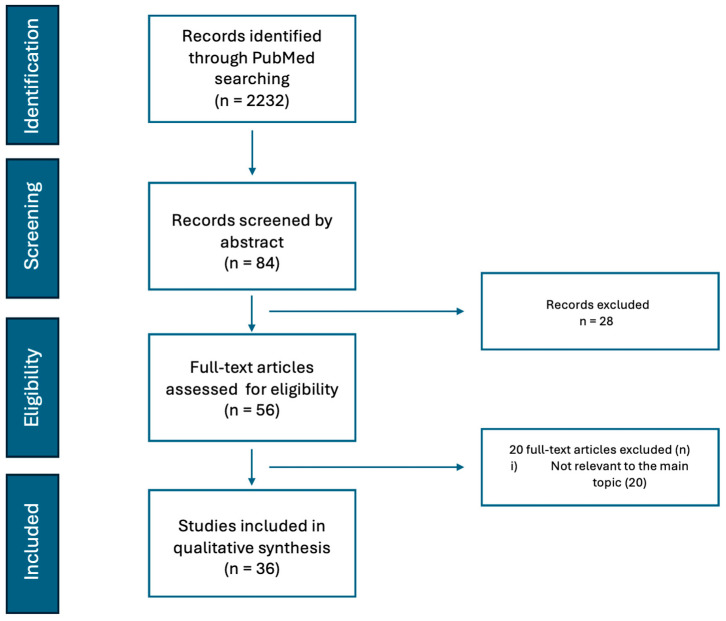
Flowchart, adapted from PRISMA, illustrating the selection process for studies on minimally invasive surgical techniques in adenomyosis and endometriosis.

A Narrative Review was conducted through a search in Pubmed, covering the period from January 2018 to 31 August 2024. We used the following query: ((Adenomyosis OR Endometriosis)) AND ((minimally invasive surgery) OR (novel surgical technique) OR (robotic surgery) OR (hysteroscopy) OR (laparoscopy)) AND (1 January 2018 [Date—Publication]: 31 August 2024 [Date—Publication]). The inclusion criteria were limited to publications in English, randomized controlled trials (RCTs), non-randomized studies (NRSs), and observational retrospective or prospective studies in surgical novel techniques in endometriosis or adenomyosis. The exclusion criteria were articles not related to the topic, surgical techniques already studied and established, single case reports, literature reviews, commentaries, and video articles (Figure 1). Duplicated articles were removed.

## 3. Results

A total of 36 articles were selected and included in this narrative review (Table 1).

### 3.1. Endometriosis

#### 3.1.1. Laparoscopic Surgical Techniques

Recent advancements in laparoscopic surgery for endometriosis have introduced new techniques to improve precision and outcomes. Transvaginal Hydro Laparoscopy (THL) is a minimally invasive procedure that enables endoscopic examination of the female pelvis. The study by Gordts et al. demonstrated that THL allows for precise diagnosis of early-stage peritoneal and ovarian endometriosis, with the added benefit of enabling treatment that causes minimal tissue damage [17].

Yamamoto et al. demonstrated that total laparoscopic retrograde hysterectomy (TLreH) is a feasible and safe approach for treating severe endometriosis with an obliterated cul-de-sac and can be safely performed even on large uteri (≥600 g) affected by this condition [18].

Regarding symptomatic rectovaginal endometriosis (RVE), Turco et al. compared two suturing directions—horizontal and vertical—for closing the posterior vaginal defect in women with endometriosis involving vaginal mucosal infiltration. They concluded that horizontal suturing of the posterior vaginal fornix defect may be associated with a higher frequency of severe postoperative complications and less effective pain control [19].

Several techniques have been developed for endometriomas-related surgery and adhesion prevention. Chaichian et al. compared Endogel^®^ application with ovarian suspension and observed that Endogel^®^ showed superior adhesion reduction within three months, although pre- and postoperative comparisons were not statistically significant [20]. Torres-de la Roche introduced a technique to preserve ovarian reserve by covering the endometrioma wound with modified polysaccharide powder for hemostasis and adhesion prevention, showing an 85% reduction in significant adhesions at follow-up [21]. Keckstein et al. proposed using HybridAPC (APC ErbeJet2 waterjet surgery system, both Erbe Elektromedizin GmbH, Tübingen, Germany) in laparoscopic surgery, which offers a promising safe method with easy handling and low adhesion rates [22]. Surgicel® has shown effectiveness in reducing recurrence rates and serves as a viable alternative to traditional cystectomy during laparoscopic drainage, minimizing its impact on ovarian reserve. Initially used for bleeding control, Surgicel^®^ was also found to lower endometrioma recurrence during follow-up [23]. Lockyer et al. demonstrated that plasma energy could serve as a promising alternative to cystectomy, achieving comparable outcomes in terms of pregnancy and recurrence rates, although further research is needed to confirm these findings [24]. Additionally, the study by Choi et al. suggests that hemostatic sealants may offer an alternative approach to bipolar coagulation for preserving ovarian reserve following laparoscopic ovarian cystectomy for endometriosis [25]. In a randomized controlled second-look clinical trial, the use of the novel adhesion barrier 4DryField® PH reduced adhesion formation by 85%. This barrier is a powder derived from purified potato starch that transforms into a gel when combined with a saline solution. The gel serves as a temporary physical barrier between surgically traumatized peritoneal surfaces, facilitating mesothelial healing before it is absorbed. When used as a powder, it also functions as a hemostat, which may contribute to its effectiveness in preventing adhesions, as the formation of polyfibrin mesh between surgical trauma sites and adjacent tissues is key to adhesion development [26].

The study by Noh et al. evaluated the effectiveness of a temperature-sensitive adhesion barrier in preventing port-site adhesions after single-port access (SPA) laparoscopic surgeries. The results showed that applying the barrier beneath the umbilical port significantly reduced postoperative adhesions in the periumbilical area without increasing complications, such as wound dehiscence or surgical site infections [27]. The study by Thabet et al. showed that pulsed high-intensity laser therapy effectively alleviates pain, reduces adhesions, and enhances quality of life in women with endometriosis [28]. Lastly, in the study by Crestani et al., laparoscopic sclerotherapy for endometriomas larger than 40 mm, performed during surgery for deep infiltrating endometriosis, demonstrated minimal impact on Anti-Mullerian Hormone (AMH) levels and was found to preserve fertility and help prevent recurrence [29].

Regarding deep infiltrative endometriosis, in particular, colorectal endometriosis, a randomized controlled trial compared laparoscopic Natural Orifice Specimen Extraction (NOSE) to conventional laparoscopic colorectal resection and concluded that NOSE Colectomy is a viable surgical option for the treatment of patients with rectal deep endometriosis, although no statistically significant differences in mid-term digestive or pain outcomes were found [30]. Grigoriadis et al. also studied the NOSE approach for patients undergoing segmental bowel resection due to colorectal endometriosis. They employed Firefly F imaging with indocyanine green to verify anastomosis both before and after its completion. They concluded that, when performed by experienced surgeons, this technique is reproducible and effective for carefully selected patients [31]. Although surgery is the gold standard treatment for pain refractory to medical management or partial occlusion owing to rectosigmoid endometriosis, surgical resection can be associated with major perioperative complications. Compared to general surgery practices, intraoperative proctosigmoidoscopy has proven to be a safer and more effective technique. It allows direct inspection of anastomosis from within, reducing the risk of complications associated with intestinal anastomosis after segmental resection [32]. 

Transumbilical single-port laparoscopy (LESS) is commonly used in gynecological surgery; however, its use in treating deep infiltrating endometriosis (DIE) is limited due to its complexity and specific challenges. The study by Zhang et al. suggests that LESS for DIE, based on retroperitoneal pelvic spaces anatomy, may be safe and feasible. This approach simplifies the surgery, shortens the procedure time, and minimizes blood loss and complications [33].

Soares et al. assessed the feasibility and risk–benefit balance of performing systematic nerve-sparing surgery, including full dissection of the inferior hypogastric and afferent pelvic splanchnic nerves, during deep infiltrating endometriosis (DIE) procedures, and showed an immediate improvement of postoperative urinary outcomes in posterior DIE surgeries [34]. Laparoscopic nerve-sparing modified radical hysterectomy, with or without robotic assistance, also appears to be a safe and feasible option that offers long-term symptom relief for patients undergoing hysterectomy for various indications, including endometriosis [35].

Regarding peritoneal endometriosis, Lier et al. aimed to identify which enhanced laparoscopic imaging techniques improved the detection of peritoneal endometriosis. The results showed that 3D white-light imaging significantly increased sensitivity compared to conventional 2D white-light imaging while maintaining similar specificity. The highest sensitivity for detecting endometriotic lesions was achieved by combining NBI (narrow-band imaging) with 3D white-light imaging. However, using NBI or NIR-ICG (near-infrared indocyanine green) alone was less effective, with reduced specificity and sensitivity rates, respectively [37]. Ma et al. also showed that using NBI during laparoscopy to investigate pelvic pain improves the detection of suspected additional areas of endometriosis following a white-light examination [36].

Regarding recent technological advances, the AutoLapTM system (MST, Netanya, Israel) aims to overcome challenges in laparoscope control by introducing a transformative approach to camera manipulation technology. This system’s steering mechanism relies on advanced image analysis and computer-based instrument recognition. In a study by Wijsman et al., the reliability and efficiency of the core electromechanical components of the AutoLap™ system [Intuitive Surgical, Sunnyvale, CA, USA] were evaluated in a clinical setting. Usability questionnaires revealed satisfactory feedback from all surgeons across all procedures, with a median satisfaction score of 4 out of 5 [38].

#### 3.1.2. Robotic Surgical Techniques

Robot-assisted surgery (RAS) has become an increasingly popular option for the treatment of endometriosis, offering enhanced precision compared to traditional laparoscopic techniques. Several studies have been published regarding RAS techniques in endometriosis. Robot-assisted transvaginal NOTES (RvNOTES) uses the full potential of robotic surgery to improve patient outcomes. In a study by Zhang et al., surgeries using RvNOTES were successfully completed, showing a reduction in chronic pelvic pain associated with endometriosis. Notably, robotic assistance offered three-dimensional visualization and stabilized instruments, enabling meticulous resection of endometriosis and precise movements during critical anatomical dissection [41]. Xu et al. aimed to extend the use of RvNOTES for managing stage IV endometriosis during total hysterectomy, with or without complete cul-de-sac obliteration. They proposed that, in the hands of experienced surgeons, this approach is feasible [42]. The study by Guan et al. represents the first exploration of robotic single-port vNOTES hysterectomy. By combining the surgical approach of transvaginal NOTES with the robotic single-port (SP) system, they demonstrated a potential alternative surgical platform for hysterectomy, which may also be applicable in cases requiring concurrent endometriosis resection [43]. In the study by Huang et al., robotic single-site surgery (RSSS) was regarded as a safe, viable, and acceptable platform for surgically treating endometriosis across all stages (I–V) [40]. In the study by Fan et al., robotic laparoendoscopic single-site surgery (LESS) was used to treat endometriosis in 36 adolescents who failed medical management. Two surgical techniques were employed: focal resection for mild disease and butterfly resection for severe or atypical disease. Both methods proved feasible and effective in reducing pain and recurrence rates [44].

The crossed setup for the robotic instruments was developed to address certain limitations of the parallel configuration used with endowristed instruments. Yang et al. introduced an innovative robotic glove port technique (RGPT) by adapting the previously established glove port method to robot-assisted single-site surgery (RSSS). This technique utilizes a parallel arrangement of endowristed rigid instruments, incorporating both coaxial and chopstick methods. It is easily performed via transvaginal and transabdominal approaches [45].

Fluorescence-guided surgery is used to improve the detection of endometriotic lesions during surgery using fluorescent dyes, such as indocyanine green (ICG). In the study by Delgadillo Chabolla et al., there were no complications with ICG while identifying and minimizing urinary tract injury during the surgical resection of endometriosis via robotic transvaginal natural orifice transluminal endoscopy surgery (RvNOTES) [46].

RAS can also be a feasible approach to DIE. In the study by Giannini et al., robotic surgery for deep infiltrating endometriosis of the ureter was feasible and enabled the complete resection of ureteral nodules [47]. Regarding diaphragmatic endometriosis (DE), robotic treatment of severe DE, when performed by experts, proved to be feasible, effective, and safe [53].

In addition, new robotic systems have recently emerged in the market, including the Medtronic Hugo™ RAS system (Medtronic, Minneapolis, MN, USA©) [54]. The applicability to gynecology is demonstrated in a study by Olsen et al., in which eleven patients underwent gynecological procedures for endometriosis. All procedures were completed successfully with no complications during surgery [39].

### 3.2. Adenomyosis

#### 3.2.1. Laparoscopic Surgical Techniques

Due to the growing emphasis on fertility preservation in adenomyomectomy, the demand for laparoscopic adenomyomectomy has risen. Sun et al. reported a novel conservative surgical procedure for adenomyosis, combining laparoscopic adenomyomectomy with intraoperative placement of levonorgestrel-releasing intrauterine device (LNG-IUS) [48]. Jiang et al., in a three-year follow-up, concluded that this procedure showed a lower recurrence rate for adenomyosis and proved more effective than surgery alone in treating symptoms such as menorrhagia and dysmenorrhea [49].

The study by Shunshi et al. showed that transvaginal ultrasound and laparoscopy-guided percutaneous microwave ablation (TLPMA) (ECO-100, Yigao Microwave System Corp., Nanjing, China) is a minimally invasive alternative treatment for adenomyosis, offering a low-risk approach with a quick recovery and minimal side effects. It significantly reduces the uterine and lesion volumes, with good long-term results [50].

#### 3.2.2. Robot-Assisted Surgical Techniques

Adenomyomectomy through robotic surgery presents several advantages. Despite the indistinct border between normal and adenomyotic tissue, the 3D magnified vision enables clear differentiation between the two types of tissue. In addition, the instruments’ seven degrees of motion facilitate the effective closure of the deep spaces created during this procedure. Hijazi et al. proposed that closing myometrial defects in a layer-by-layer manner after robot-assisted laparoscopic adenomyomectomy is a viable approach. This technique may also help preserve myometrial alignment and maintain sufficient uterine wall thickness post-adenomyomectomy [51].

#### 3.2.3. Other Techniques

Philip et al. demonstrated that NovaSure^®^ (Hologic, Inc., Marlborough, MA, USA) provides effective relief from painful and bleeding symptoms associated with adenomyosis in both the short term and the long term, although its effectiveness in managing bleeding may gradually decrease over time. Despite this, NovaSure^®^ remains a viable alternative to hysterectomy, particularly for patients nearing menopause [52].

## 4. Discussion

Endometriosis and adenomyosis are complex diseases that need specialized care. The evolution of surgical techniques in this field reflects ongoing efforts to optimize outcomes, reduce disease recurrence, and enhance patients’ quality of life. In recent years, advances in minimally invasive techniques, as well as precision-driven approaches, have improved precision and reduced the risk of complications associated with traditional open surgeries. Furthermore, fertility-preserving options, such as conservative excision, have become more viable due to these advancements. These innovative techniques not only target symptom relief but also address underlying pathologies, offering long-term relief and a more tailored approach to patient care [55].

As previously mentioned, the use of RAS is increasing and being applied at various stages of endometriosis, including deep infiltrative endometriosis (DIE) and even simple hysterectomies. The introduction of robot-assisted surgery has opened new possibilities, particularly for complex cases involving DIE. Robotic surgery offers improved visualization, greater dexterity, and more precise dissection skills compared to conventional laparoscopy. Until recently, the DaVinci^®^ Surgical System (Intuitive Surgical, Sunnyvale, CA, USA) was the only robotic system available. However, several new robotic systems have entered the market since the introduction of the Senhance^®^ Telerobotic system (Asensus Surgical^®^ Inc., Durham, NC, USA) in late 2016 and the Medtronic Hugo^TM^ RAS system in 2021 [8,9,39,56]. Studies have shown that RAS is not clinically inferior to standard laparoscopy, although it typically requires a longer operating time. Despite this, there is no significant difference between the two methods in blood loss, complication rates, or conversion rates. RAS has proven to be safe and effective in treating endometriosis, particularly in complex cases involving deep infiltrating endometriosis affecting the bowel or urinary tract. In fact, a recent study has shown the Da Vinci^®^ system and Hugo^TM^ RAS offer comparable performances. However, most supporting research has been retrospective, and successful outcomes depend heavily on the surgeon’s expertise [57,58].

The high cost of the procedure is often mentioned as a disadvantage of robotic surgery. Nevertheless, robot-assisted surgery may offer benefits in more challenging surgeries, such as those involving deep pelvic structures or multiple organ systems. However, large-scale studies are needed to confirm whether robotic surgery improves long-term outcomes beyond what can be achieved with advanced laparoscopic techniques and to determine which robotic system is better. The development of new technological systems is also encouraged [8,9,39].

New surgical approaches, such as RAS, require practice and time to learn them. Some strategies have been developed to simplify the learning process, such as training programs for surgeons, for example, a standardized robot-assisted hysterectomy performed by a resident under the supervision of a senior surgeon using a dual-console system [59].

In contrast to endometriosis, adenomyosis presents distinct challenges due to its heterogeneity and the limited treatment options available, particularly in cases where preserving fertility is a priority. Complete excision of adenomyosis can be challenging due to the absence of a distinct cleavage plane, and small residual lesions may persist, increasing the risk of recurrence [48,60]. Surgical treatment options are more limited, particularly for women who desire to preserve fertility. Therefore, newer techniques such as localized adenomyomectomy have been developed [49,60,61]. Robotic adenomyomectomy is a viable option for women with adenomyosis, showing surgical outcomes comparable to those achieved with the laparoscopic approach [62]. Compared to laparoscopy, robotic visualization led to the detection of more confirmed lesions across all anatomical locations. However, large-scale long-term studies are required to validate these findings in diverse clinical settings and to assess whether 3D robotic resection, as opposed to 2D laparoscopic resection, positively impacts symptom relief, recurrence rates, and fertility outcomes [63].

The lack of randomized controlled trials comparing treatment strategies emphasizes the need for more research to identify the best management approaches. In fact, most studies are retrospective and have short follow-up periods and small sample sizes. In addition, several techniques have been developed and published as single case studies or video articles, which were not included in this study. Therefore, comparative and randomized studies could be conducted in the future, including other multicenter prospective studies using standardized surgical procedures.

The combination of surgery with adjuvant medical therapy has become a key strategy in both endometriosis and adenomyosis management. In many cases, hormonal treatments such as oral contraceptives methods are used before or after surgery to suppress disease activity and reduce the size of lesions [49,60]. In this research, we also found that the combination of laparoscopic adenomyomectomy with the intraoperative placement of a levonorgestrel-releasing intrauterine device (LNG-IUS) resulted in a lower recurrence rate of adenomyosis and proved more effective in relieving symptoms compared to surgery alone [48]. This combined approach is particularly valuable in cases where extensive disease involvement makes complete excision difficult.

For women of childbearing age, preserving ovarian function is paramount, and careful management of adhesions is critical for maintaining reproductive potential [64]. Additionally, approximately 30 to 50% of women with endometriosis have infertility and primary surgery may increase postoperative pregnancy rates [7,65,66]. As previously described, several strategies have been studied to reduce the risk of adhesions and ovarian lesions. Extended follow-up periods and comparative studies are essential for assessing treatment efficacy and evaluating their impact on long-term clinical outcomes more accurately [20].

Regarding future directions, endometriosis and adenomyosis will still require not only a multidisciplinary approach with specialized and experienced teams but also novel techniques tailored to individual patient needs. This is especially relevant in complex cases of deep infiltrating endometriosis or adenomyosis. Despite extensive research, some aspects remain controversial, highlighting the need for prospective studies to refine treatment strategies and enhance patient outcomes. The advancements in surgical techniques and technology will lead to several novel surgical techniques, and these innovations will reshape surgery, offering patients more effective, safer, and less invasive treatment options.

## 5. Conclusions

These novel surgical approaches are becoming more precise and less invasive. Newer tools like robotic surgery are expanding treatment options. As technology continues to evolve, the focus remains on improving long-term outcomes, reducing recurrence rates, improving fertility preservation, and enhancing the quality of life of these patients. Multidisciplinary care combining surgery with medical therapy and personalized surgical planning will improve long-term outcomes.

## Figures and Tables

**Table 1:** Main characteristics of the included studies.

Endometriosis					
Type of Surgery	Surgical Technique	Author and Year	Type of Study	Number of Subjects	Conclusions
Laparoscopy	Transvaginal Hydro Laparoscopy (THL) [17]	Gordts et al., 2023	Retrospective Multicenter Cohort Study	2288	THL enabled precise diagnosis of early-stage peritoneal and ovarian endometriosis, allowing for treatment with minimal tissue damage [17].
	Total laparoscopic retrograde hysterectomy (TLreH) for severe endometriosis with obliterated cul-de-sac [18]	Yamamoto et al., 2021	Retrospective Case-control Study	92	TLreH proved to be a feasible and safe approach for severe endometriosis with an obliterated cul-de-sac [18].
	Comparison between suturing the posterior vaginal breach horizontally and vertically [19]	Turco et al., 2024	Retrospective Multicenter Cohort Study	101	Horizontal suturing of the posterior vaginal fornix defect may be linked to a higher incidence of severe postoperative complications and poorer pain management outcomes [19].
	Hyaluronic acid gel versus ovarian suspension [20]	Chaichian et al., 2022	Randomized Clinical Trial	50	Compared to ovarian suspension, HYAcorp Endogel^®^ may effectively reduce the risk of adhesions three months post-surgery [20].
	Endometrioma stripping technique with a plant-based medical product with high-hemostatic and anti-adhesion properties [21]	Torres-de la Roche et al., 2020	Retrospective Cohort Study	10	Preliminary results indicate that endometrioma stripping followed by the application of a polysaccharide agent, serving as both a hemostatic and anti-adhesion solution, may offer an ovary-sparing approach in infertility surgeries for endometrioma [21].
	Hybrid argon plasma coagulation (HybridAPC) [22]	Keckstein et al., 2022	Prospective Randomized Clinical Trial	39	It highlights the feasibility of the surgical technique, showing promising potential in preventing adhesions. Compared to sharp excision, HybridAPC appears to be a safe, tissue-preserving, and efficient approach for treating peritoneal endometriosis [22].
	Surgicel^®^ [23]	Shaltout et al., 2019	Prospective Randomized Clinical Trial	215	The use of Surgicel during cystectomy or drainage of endometriomas was generally safe, with no significant complications or side effects reported [23].
	Plasma energy [24]	Lockyer et al., 2019	Retrospective Cohort Study	21	Plasma energy offers a promising alternative to stripping cystectomy, yielding comparable outcomes in terms of postoperative pregnancy rates and recurrence [24].
	Hemostatic sealants [25]	Choi et al., 2018	Randomized Prospective Data Collection	80	Hemostatic sealants (such as FloSeal® or TachoSil^®^) could serve as an alternative to bipolar coagulation for preserving ovarian reserve following laparoscopic ovarian cystectomy for endometriosis [25].
	Adhesion barrier 4DryField^®^ [26]	Krämer et al., 2021	Prospective Randomized Controlled Clinical Trial	50	The application of the adhesion barrier 4DryField^®^ PH could reduce adhesion formation by as much as 85% [26].
	Temperature-sensitive adhesion barrier in single-port access (SPA) laparoscopic surgeries [27].	Noh et al., 2021	Prospective Cohort Study	37	Applying the barrier beneath the umbilical port significantly reduced postoperative adhesions in the periumbilical area without increasing complications [27].
	Pulsed High Energy [28]	Thabet et al., 2018	Randomized Controlled Trial	40	Pulsed high-intensity laser therapy is an effective treatment for pain relief, reducing adhesions, and enhancing the quality of life in women with endometriosis [28].
	Sclerotherapy [29]	Crestani et al., 2023	Retrospective Cohort Study	69	In patients with deep pelvic endometriosis, particularly those with large endometriomas and a desire for pregnancy, laparoscopic sclerotherapy may be a valuable alternative to cystectomy [29].
	Laparoscopic Natural Orifice Specimen Extraction (NOSE) Colectomy [30]	Dobó et al., 2023	Single-center Randomized Controlled Trial	91	Both NOSE and conventional laparoscopic colectomy are safe methods for the surgical treatment of colorectal deep endometriosis. The occurrence of long-term bowel dysfunction does not appear to be related to a specific surgical technique [30].
	NOSE colectomy for colorectal endometriosis [31]	Grigoriadis et al., 2022	Retrospective Cohort Study	50	Both NOSE and conventional laparoscopic colectomy are safe approaches for the surgical treatment of colorectal deep endometriosis. Long-term bowel dysfunction does not seem to be associated with a particular surgical technique [31].
	Intraoperative proctosigmoidoscopy [32]	Raimondo et al., 2024	Prospective Multicentric Cohort Study	28	Intraoperative proctosigmoidoscopy appears to be feasible and time-efficient for women undergoing discoid resection for rectosigmoid endometriosis, alongside standard integrity tests [32].
	Transumbilical single-port laparoscopy (LESS) for DIE [33]	Zhang et al., 2023	Retrospective Case Series Study	33	LESS for DIE, based on retroperitoneal pelvic anatomy, is a safe and feasible approach that simplifies surgery, reduces blood loss, and minimizes complications [33].
	Systematic nerve-sparing surgery [34]	Soares et al., 2021	Single-center Observational Before-and-after Study	121	Systematic and complete nerve sparing, including pelvic splanchnic nerve dissection, during surgery for posterior DIE improves immediate postoperative urinary outcomes, reducing the need for self-catheterization without increasing operating time or complication rates [34].
	Laparoscopic nerve-sparing modified radical hysterectomy with or without robotic assistance [35]	Nezhat et al., 2020	Retrospective Observational Study	112	Laparoscopic nerve-sparing modified radical hysterectomy, with or without robotic assistance, is a safe and feasible alternative that offers long-term symptom relief for patients undergoing hysterectomy for various indications [35].
	Narrow-band imaging (NBI) [36]	Ma et al., 2019	Prospective Cohort Trial	53	The use of NBI during laparoscopy for investigating pelvic pain is helpful in identifying additional areas of endometriosis when it is already suspected following a white-light survey [36].
	Enhanced laparoscopic imaging techniques [37]	Lier et al., 2020	Prospective Single-center Randomized Clinical Trial	20	Enhanced laparoscopic imaging with 3D white light, combined with NBI, increases the detection of peritoneal endometriosis compared to conventional 2D white-light imaging. These advanced techniques allow for a more thorough laparoscopic resection of endometriosis [37].
	The AutoLap^TM^ system: an image-based robotic camera steering device [38]	Wijsman et al., 2017	Prospective Multicenter Study	66	The AutoLap™ system is an effective, safe, and user-friendly solution for robotic camera control during a range of abdominal procedures [38].
Robotics	Hugo^TM^ robot-assisted surgery system [39]	Olsen et al., 2024	Retrospective Cohort Study	9	The Medtronic Hugo^TM^ RAS system is safe and feasible for robot-assisted surgery for endometriosis [39].
	Robotic single-site surgery with optional additional port [40]	Huang et al., 2021	Retrospective Cohort Study	334	RSSS is a safe, effective, and viable platform for the surgical treatment of endometriosis at all stages [40].
	Robot-assisted vaginal natural orifice transluminal endoscopic surgery (RvNOTES) [41]	Zhang et al., 2021	Retrospective Case Series Study	33	RvNOTES is a feasible approach for treating endometriosis, showing effective and safe short-term outcomes. Robotic assistance enhances 3D visualization and instrument stability, allowing for precise endometriosis resection and careful anatomical dissection during vNOTES procedures [41].
	RvNOTES with total hysterectomy for management of stage IV endometriosis [42]	Xu et al., 2024	Retrospective Case Series Study	33	RvNOTES may be a feasible surgical approach for treating stage IV endometriosis, including cases with complete obliteration of the cul-de-sac, when performed by experienced surgeons [42].
	Robotic Single-Port Transvaginal NOTES (RSP-vNOTES) hysterectomy [43]	Guan et al., 2024	Retrospective Case Series Study	28	RSP-vNOTES is a new single-port surgical technique and a promising alternative for vaginal surgeries [43].
	Robotic single-site surgery techniques for adolescent endometriosis: focal versus butterfly [44]	Fan et al., 2021	Retrospective Comparative Study	32	Both focal resection and butterfly resection robotic LESS techniques are feasible and effective in alleviating pain and reducing recurrence in adolescents with endometriosis [44].
	Robotic glove port technique (RGPT) for the endowristed rigid instruments in robotic single-site transabdominal and transvaginal surgery [45]	Yang et al., 2020	Retrospective Cohort Study	35	RSS surgery using the RGPT is a safe and feasible technique, easily performed via both transvaginal and transabdominal routes with endowristed rigid instruments. This approach could be a valuable addition to the gynecologic surgeons’ armamentarium for robotic reconstructive and fertility-preserving procedures, including myomectomy, adenomyomectomy, ovarian cystectomy, and transvaginal NOTES hysterectomy [45].
	Indocyanine green (ICG) in robotic transvaginal natural orifice transluminal endoscopy surgery (RvNOTES) [46]	Delgadillo Chabolla et al., 2024	Retrospective Multicenter Case Series Study	53	ICG fluorescence in RvNOTES seems to be a safe technique for ureteral localization and preservation [46].
	Robotic surgery for deep infiltrating endometriosis of the ureter [47]	Giannini et al., 2018	Retrospective Case Series Study	31	Robotic surgery for deep infiltrating endometriosis of the ureter was feasible and allowed complete resection of ureteral nodules in all cases [47].
Adenomyosis					
Type of surgery	Surgical technique	Author and year	Type of study	Number of subjects	Conclusions
Laparoscopy	Laparoscopic adenomyomectomy with intraoperative placement of levonorgestrel-releasing intrauterine device (LNG-IUS) [48]	Sun et al., 2021	Case Series Study	52	Laparoscopic adenomyomectomy, combined with intraoperative insertion of the LNG-IUS, is an innovative and effective conservative surgical approach for treating symptomatic adenomyosis [48].
	Laparoscopic adenomyomectomy combined with levonorgestrel-releasing intrauterine system [49]	Jiang et al., 2024	Retrospective Comparative Study	139	A combination of surgery and LNG-IUS treatment was more effective than surgery alone in managing adenomyosis-related symptoms, such as menorrhagia and dysmenorrhea. It provided better symptom control and reduced recurrence rates [49].
	Transvaginal ultrasound and laparoscopy-guided percutaneous microwave ablation (TLPMA) [50]	Shunshi et al., 2023	Prospective Cohort Study	79	TLPMA is a feasible and minimally invasive approach for treating adenomyosis, effectively reducing uterine and lesion volumes with favorable long-term outcomes [50].
Robotics	Myometrial defect closure after robot-assisted laparoscopic adenomyomectomy [51]	Hijazi et al., 2021	Retrospective Cohort Study	34	A layer-by-layer closure of myometrial defects following robot-assisted laparoscopic adenomyomectomy is a reproducible uterine-conserving technique. This method effectively reduces pain while preserving myometrial wall thickness, ensuring proper alignment of uterine layers and endometrial integrity [51].
Other techniques	NovaSure® global endometrial ablation [52]	Philip et al., 2018	Single-center Longitudinal Cohort Study	43	NovaSure® can effectively manage the painful and heavy bleeding symptoms associated with adenomyosis in both the short term and the long term. However, bleeding may worsen over time. Despite this, it continues to be a viable alternative to hysterectomy, especially for patients nearing menopause [52].

## Data Availability

All data analyzed in this article are included in the manuscript and are available in a publicly accessible repository. Further inquiries can be directed to the corresponding author.
